# Effects of *Schizolobium parahyba* Extract on Experimental Bothrops Venom-Induced Acute Kidney Injury

**DOI:** 10.1371/journal.pone.0086828

**Published:** 2014-02-14

**Authors:** Monique Silva Martines, Mirian M. Mendes, Maria H. M. Shimizu, Veridiana Melo Rodrigues, Isac de Castro, Sebastião R. Ferreira Filho, Denise M. A. C. Malheiros, Luis Yu, Emmanuel A. Burdmann

**Affiliations:** 1 LIM 12, Division of Nephrology, University of Sao Paulo Medical School, São Paulo, São Paulo, Brazil; 2 Biological Science Institute, Goias Federal University, Jataí, Goiás, Brazil; 3 Institute of Genetics and Biochemistry, Uberlandia Federal University, Uberlândia, Minas Gerais, Brazil; 4 Division of Nephrology, University of Sao Paulo Medical School, São Paulo, São Paulo, Brazil; 5 Division of Nephrology, Uberlandia Federal University, Uberlândia, Minas Gerais, Brazil; National Institutes of Health, United States of America

## Abstract

**Background:**

Venom-induced acute kidney injury (AKI) is a frequent complication of *Bothrops* snakebite with relevant morbidity and mortality. The aim of this study was to assess the effects of *Schizolobium parahyba* (SP) extract, a natural medicine with presumed anti-Bothrops venom effects, in an experimental model of *Bothrops jararaca* venom (BV)-induced AKI.

**Methodology:**

Groups of 8 to 10 rats received infusions of 0.9% saline (control, C), SP 2 mg/kg, BV 0.25 mg/kg and BV immediately followed by SP (treatment, T) in the doses already described. After the respective infusions, animals were assessed for their glomerular filtration rate (GFR, inulin clearance), renal blood flow (RBF, Doppler), blood pressure (BP, intra-arterial transducer), renal vascular resistance (RVR), urinary osmolality (UO, freezing point), urinary neutrophil gelatinase-associated lipocalin (NGAL, enzyme-linked immunosorbent assay [ELISA]), lactate dehydrogenase (LDH, kinetic method), hematocrit (Hct, microhematocrit), fibrinogen (Fi, Klauss modified) and blinded renal histology (acute tubular necrosis score).

**Principal Findings:**

BV caused significant decreases in GFR, RBF, UO, HcT and Fi; significant increases in RVR, NGAL and LDH; and acute tubular necrosis. SP did not prevent these changes; instead, it caused a significant decrease in GFR when used alone.

**Conclusion:**

SP administered simultaneously with BV, in an approximate 10∶1 concentration, did not prevent BV-induced AKI, hemolysis and fibrinogen consumption. SP used alone caused a decrease in GFR.

## Introduction

Snakebite envenomation, recognized as a neglected tropical disease (NDT) by the World Health Organization (WHO) since 2009 [1], is an important public health issue in several developing tropical countries [2]–[5]. In fact, venomous snakebites carry an annual mortality higher than that found in infectious diseases classically recognized as NDTs, such as schistosomiasis and leishmaniasis. In addition, venomous snakebites provoke significant morbidity and disability in young, previously healthy agricultural workers who are primarily male, resulting in a relevant socioeconomic impact on agricultural communities [6], [7].

Snake envenoming is strongly associated with poverty and is inversely related to the country’s expenditure on health care, which potentiates its middle- and long-term socioeconomic effects [8], [9]. The latest estimation of the global burden of venomous snakebite accidents and deaths used data from 135 and 162 countries, respectively, and reported that 421,000 to 1,841,000 cases of envenoming and 20,000 to 94,000 deaths occur annually. South and Southeast Asia, sub-Saharan Africa and Latin America, especially Central and South America, were the primarily affected areas [10]. Nevertheless, those numbers likely underestimate the full burden of venomous snakebites. Indeed, nationwide studies in India and Bangladesh found that the snakebite envenoming country mortality rates were approximately three times higher than the rates reported in the previously referenced manuscript [11], [12].

In Latin America, snakes of the genus *Bothrops* are the main cause of venomous snake accidents; the most clinically relevant species are *Bothrops asper, Bothrops atrox* and *Bothrops jararaca* [13], [14].

*Bothrops* venom (BV) is a complex mixture of proteins (enzymes, non-enzyme toxins and non-toxic proteins), which constitute the major part of the venom, and carbohydrates, lipids, metals, biogenic amines, free amino acids and nucleotides. The venom has proteolytic, coagulant, hemorrhagic and direct nephrotoxic activities, which together cause local injury at the bite site, ranging from an inflammatory response to severe tissue necrosis and systemic manifestations, such as coagulation system disturbances that may evolve to hemorrhagic manifestations, hypotension and acute kidney injury (AKI) [13], [15], [16]. Several toxins contribute to the clinically relevant effects of the venom, including bradykinin-releasing enzymes, phospholipases A_2_ (PLA_2_), serine proteinases, metalloproteinases, l-amino oxidases, hyaluronidases, thrombin-like enzymes, factor X and prothrombin activators, toxins that stimulate or inhibit platelet function, hemorrhagins, phosphodiesterases and 5′-nucleotidases [15].

AKI is a frequent complication after Bothrops envenoming [13], [16] and is considered the main cause of death in patients who survive the first effects of the venom [13], [14], [17]. The mechanisms causing *Bothrops* venom-induced AKI are likely high venom concentration at the renal tissue, direct venom action on the tubular cells, hemoglobinuria, glomerular deposition of fibrin microthrombi, renal vasoconstriction, release of alarmins and oxidative stress [18]–[25].

Currently, the only specific treatment against snake envenomation is the parenteral administration of antivenom constituted by a hyperimmune globulin from an animal (usually a horse or a sheep) inoculated with the selected venom [26], [27]. Early specific antivenom administration has demonstrated efficacy against snake venom-induced AKI in experimental models [20], [28], and late antivenom administration has been associated with an increased odds ratio for developing AKI in the clinical setting [16], [29]–[31]. However, the current anti-venom therapy has several limitations. It does not prevent snake venom-induced local injury, can be relatively expensive, is vulnerable to shortages, can cause hypersensitivity reactions and may be hard to find in particular areas [13], [32]–[34]. These limitations of the current animal-derived polyclonal antivenom have inspired a search for feasible alternatives, including the use of medicinal plants from traditional medicine that are normally utilized by healers on different continents [35].

*Schizolobium parahyba* (subfamily Caesalpinoideae) is a leguminous tree found from Mexico to southern Brazil. It grows in tropical forests and has been planted extensively in reforestation projects from the Amazon Basin south to São Paulo [36]. SP is popularly known as faveira, guapuruvú or umbela, and healers and shamans have traditionally used SP infusions as natural medicines for treating snakebite envenoming [37]–[39]. In Brazil, healers traditionally use leaves from the *Schizolobium parahyba* tree to treat venomous snakebite accidents. Experimental studies have shown that gross aqueous extract and flavonoids from *Schizolobium parahyba* protect against some of the effects of Bothrops venom in rodent animal models and in vitro preparations [37]–[39].

The aim of the present study was to assess the effects of the aqueous extract from *Schizolobium parahyba* (SP) in a model of *Bothrops* venom-induced AKI.

## Methods

### Animals and Diet

Male Wistar rats (250 to 300 g) from the Central Animal Facility of the University of São Paulo Medical School were housed in a temperature- and light-controlled environment. They received a standard salt and protein diet (22% protein and 1.3% sodium chloride, Nuvilab CR1, Nuvital®, PR-Brazil). They were allowed free access to tap water.

### Ethics Statement

All experiments were performed in compliance with the Brazilian Law for the Protection of Animals, and the University of São Paulo Medical School Research Ethical Committee (process n ° 191/11) approved the experimental protocol.

### *Bothrops Jararaca* Venom (BV)

Lyophilized BV was a generous gift from Butantan Institute, São Paulo, Brazil (lot 01/08–01). This BV lot, made from a pool of venom from several snakes, was divided in aliquots of 0.0025 g kept at −20°C. On the experiment day, the aliquot was dissolved in 10 ml of 0.9% saline for immediate use. Rats received 0.25 mg/kg of BV via the jugular vein at a rate of 0.02 ml/min for 40 min (Compact Infusion Pump, 975, Harvard, USA).

### *Schizolobium Parahyba* Extract (SP)

The aqueous extracts prepared from the leaves of *Schizolobium parahyba* plant were a generous gift by Mirian Machado Mendes of the Animal Toxins Biochemistry and Molecular Biology Laboratory, Federal University of Uberlandia, Brazil. SP aliquots of 0.02 g were kept at −20°C. On the experiment day, SP was dissolved in 10 ml of 0.9% saline and infused via the jugular vein at a dose of 2.0 mg/kg at a rate of 0.02 ml/min for 40 min (Compact Infusion Pump, 975, Harvard, USA). This dose was selected from previous publications (37–39).

### Treatment Groups

Animals were divided into four groups:

1. Control (C): received 1.6 ml of 0.9% saline infusion via the jugular vein for 80 min at a rate of 0.02 ml/min.

2. BV: received 0.25 mg/kg of *Bothrops jararaca* venom infusion via the jugular vein for 40 min at a rate of 0.02 ml/min followed by 0.8 ml of 0.9% saline infusion for 40 min at a rate of 0.02 ml/min.

3. SP: received 0.8 ml of 0.9% saline infusion via the jugular vein for 40 min at a rate of 0.02 ml/min followed by 2.0 mg/kg of *Schizolobium parahyba* aqueous extract for 40 min at a rate of 0.02 ml/min.

4. Treatment (T): received sequential infusions of BV and SP as described above. SP was infused immediately after BV infusion.

### Glomerular Filtration Rate (Inulin Clearance)

Animals were anesthetized with an intraperitoneal injection of thiopental (40 mg/kg). Then, a tracheotomy was performed, and polyethylene tubes (PE-50) were placed in the carotid artery both to monitor MAP and for blood collection and in the right and left jugular veins for 0.9% saline, inulin solution, BV and SP infusion. The carotid MAP was continuously measured with an electronic transducer connected to a digital polygraph (BIOPAC Systems, Inc., Santa Barbara, California, USA) throughout the experiments. A polyethylene tube (PE-160) was placed and sutured into the bladder. Animals were maintained at a stable temperature (36.5±1°C) by a thermostatically controlled warming table during the experiment. After surgery, each animal received a loading dose of 10 mg/kg of inulin solution (Sigma Chemical Co., St. Louis, 100 mg of inulin diluted in 15 ml of 0.9% saline). Next, the animals were given 2 ml of 0.9% saline, which was followed by a maintenance infusion of the inulin solution at a rate of 0.055 ml/min (Compact Infusion Pump, 975, Harvard, USA). After a 10-min equilibration time, 0.9% saline, BV or SP were infused into the groups as described above. After a second 20-min equilibration time, urine was collected in pre-weighed Eppendorf vials for three successive 30-min periods. A blood sample (0.4 ml) was taken at the midpoint of each urine collection and was replaced with an equal volume of 0.9% NaCl.

The serum and urine inulin levels were measured using a colorimetric assay (spectrophotometer Femto 700 plus, Brazil). The inulin clearance values, which are expressed as ml/min/100 g body weight, represent the mean value from the three clearance periods. The results for the MAP represent the mean value of the measurements taken.

The urinary volume was assessed by the weight difference in the Eppendorf vials before and after urine collection.

### Renal Hemodynamic Study (Doppler)

Anesthesia, tracheostomy and carotid and jugular surgical preparations were performed as previously described. After these steps, a ventral midline incision was made, exposing the left renal artery, which was dissected, and a suitable probe (R series, 1.5 mm, Transonic Systems, Ithaca, NY, USA) was placed around the artery. Then, a loading injection of 5 ml 0.9% saline was given, and 0.9% saline, BV or SP were infused as already described, followed by a maintenance infusion of 0.9% saline at a rate of 0.055 ml/min (Compact Infusion Pump, 975, Harvard, USA). In sequence, ultrasonic RBF measurements were performed (T 106, Transonic Systems Inc., Ithaca, NY, USA) over a 40-min observation period (measured at four points at 10-min intervals). The RBF values represent the means of the four measurements. The MAP was continuously monitored as previously described. The RVR was calculated with the usual formula (RVR = BP/RBF).

The urinary volume was collected during 180 min for measurements of the tubular injury biomarkers KIM-1 and NGAL; sodium, potassium and creatinine levels; and osmolality. At the end of the experiment, blood samples were taken to determine the sodium, potassium, creatinine, fibrinogen and lactate dehydrogenase levels.

### Urinary Biomarkers, Biochemical Analysis, Hematocrit and Osmolality

Both biomarkers were measured by ELISA immunoenzymatic quantitative assays (KIM-1 by KIM-1 quantitative ELISA kit, RKM 100, R&D Systems, Minneapolis, USA and NGAL by KIT 046, BioPorto, Denmark).

Serum and urinary creatinine levels were assessed using the Jaffe colorimetric method (automatized analyzer, Cobas C111, Roche, Switzerland).

Serum and urinary sodium and potassium levels were assessed with an electrolyte analyzer (Celm FC-280, Brazil). The fractional excretions of sodium and potassium were calculated using the usual formulas, which are: [urinary Na/plasma Na divided by urinary Cr/plasma Cr]×100 and [urinary K/plasma K divided by urinary Cr/plasma Cr]×100.

Lactate dehydrogenase was assessed with a kinetic method using UV absorbency, which measures the conversion from L-lactate in pyruvate (automatized analyzer, Cobas C111, Roche, Switzerland).

Fibrinogen was assessed using the Clauss modified method (hemostasis coagulation analyzer, Stago Start 4, France).

Hematocrit was assessed using the microhematocrit method.

The urinary osmolality was assessed by freezing point analysis (Advanced™ Osmometer Model 3D3, USA).

### Histological Study

At the conclusion of the GFR experiments, the animals were euthanized by an intravenous injection of thiopental. Renal tissue samples were obtained and fixed in 10% buffered formalin (sodium phosphate 0.05 M, pH 7.3) for 24 h at 4°C. After 24 h, the samples were stocked in 70°C ethanol and then embedded in paraffin. For the renal histological evaluation, 3- to 4-µm-thick longitudinal sections were stained with periodic acid-Schiff (PAS). All microscopic fields in each section were examined under light microscopy at a final magnification of 250X. The presence and extent of acute tubular necrosis (ATN) was assessed via histomorphometry by a single observer, who was blinded to the treatment groups. The changes considered as indicative of ATN were brush border loss, *cytoplasmic* and nuclear integrity loss, sloughing of tubular cells into the lumen and hyaline casts in the tubular lumen.

Tubular injury was evaluated using a semiquantitative scale, in which the percentage of the cortex showing epithelial necrosis was assigned a score as follows:

Grade 0: <10% of the section affected;

Grade 1∶10% to 25% of the section affected;

Grade 2∶26% to 50% of the section affected;

Grade 3: >50% of the section affected.

### Statistical Analysis

Continuous data were tested for normality using the Shapiro-Wilks test. Parametric data are presented as means ± standard deviations and were compared using ANOVA for non-repeated data with the Student-Newman-Keuls post-test. Non-parametric data are presented as medians and 25th –75th percentiles and were compared by the Kruskal-Wallis test with the Dunn or Conover-Inman post-test. Statistical significance was set at 5% (p<0.05).

## Results

### Renal Function and Renal and Systemic Hemodynamics

Bothrops venom caused sharp, striking and statistically significant decreases in the glomerular filtration rate (GFR) and renal blood flow (RBF) and an increase in the renal vascular resistance (RVR), which were not prevented by sequential SP aqueous extract infusion in the treatment group. The infusion of SP alone caused a moderate but statistically significant decrease in GFR. The four groups had similar mean arterial pressure (MAP).

The four groups had similar urinary output. The BV and treatment groups had higher sodium fractional excretions, but the results did not reach statistical significance. The fractional excretion of potassium was similar among the groups. BV caused a significant decrease in the urinary osmolality, which was not prevented by sequential infusion of SP. The control and SP alone groups had similar urinary osmolality.

These results are summarized in Table 1.

**Table 1:** Renal function, renal and systemic hemodynamics in control (C), *Bothrops* venom (BV), *Schizolobium parahyba* aqueous extract (SP) and BV followed by SP (T) groups.

	C	BV	SP	T
GFR (ml/min/100g)	0.88±0.13	0.47±0.15 a	0.64±0.12 b	0.52±0.22 a
	(8)	(8)	(8)	(8)
RBF (ml/min)	6.4 (6.0–7.1)	3.0 (2.3–3.5)^a^^,^ c	6.3 (6.0–6.9)	3.7 (3.0–4.8)^a^^,^ d
	(10)	(10)	(10)	(10)
RVR (mmHg/ml/min)	20 (17–25)	39 (31–60)^a^^,^ c	21 (18–24)	34 (23–40)^e^^,^ f
	(10)	(10)	(10)	(10)
MAP (mmHg)	135±16	128±23	133±13	120±14
	(10)	(10)	(10)	(10)
UO (µl/min)	182±90	203±152	168±88	177±94
	(8)	(8)	(8)	(8)
FeNa (%)	0.4 (0.1–1.0)	1.4 (0.5–3.0)	0.3 (0.2–1.0)	0.8 (0.4–2.3)
	(10)	(9)	(9)	(9)
FeK (%)	23±13	36±19	20±9	28±9
	(10)	(9)	(9)	(9)
Uosm (mOsm/kg)	1,305 (1,019–1,496)	569 (530–744)^b^^,^ e	1,275 (945–1,504)	669 (468–772)^b^^,^ e
	(10)	(9)	(10)	(10)

Data are mean ± SD or median (quartiles); (n); GFR: glomerular filtration rate; RBF: renal blood flow; RVR: renal vascular resistance; MAP: mean arterial pressure; UO: urinary output; FeNa: fractional excretion of sodium; FeK: fractional excretion of potassium; Uosm: urinary osmolality.

a p<0.001 vs. control;

b p<0.01 vs. control;

c p<0.001 vs. SP;

d p<0.01 vs. SP;

e p<0.05 vs. control;

f p<0.05 vs. SP.

### Urinary Biomarkers

The BV and treatment groups had significantly higher levels of urinary neutrophil gelatinase-associated lipocalin (NGAL) compared to the control and SP groups. The four groups had similar urinary kidney injury molecule-1 (KIM-1) values.

These results are summarized in Table 2.

**Table 2:** NGAL and KIM-1 in control (C), *Bothrops* venom (BV), *Schizolobium parahyba* aqueous extract (SP) and BV followed by SP (T) groups.

	C	BV	SP	T
NGAL (µg/ml)	9.9 (6.5–18.1)	20.7 (13.9–33.2) a ^,^ b	9.8 (8.0–12.5)	18.8 (12.6–22.1) b
	(10)	(10)	(10)	(10)
KIM-1 (ng/ml)	0.46 (0.32–0.57)	0.43 (0.24–1.04)	0.35 (0.26–0.63)	0.51 (0. 25–1.17)
	(10)	(10)	(10)	(10)

Data are median (quartiles); (n); NGAL: neutrophil gelatinase-associated lipocalin; KIM −1: kidney injury molecule-1.

a p<0.05 vs. control;

b p<0.05 vs. SP.

### Biochemical Analysis and Hematocrit

The serum sodium and potassium levels were similar in the four groups. BV caused statistically significant decreases in hematocrit and fibrinogen levels and a statistically significant increase in lactate dehydrogenase (LDH), which was not prevented by sequential infusion with SP. The control and SP alone groups had similar hematocrit, fibrinogen and LDH values. These results are summarized in table 3.

**Table 3:** Hematocrit (HcT), lactate dehydrogenase (LDH), fibrinogen (Fi), sodium (Na) and potassium (K) in control (C), *Bothrops* venom (BV), *Schizolobium parahyba* aqueous extract (SP) and BV followed by SP (T) groups.

	C	BV	SP	T
Hct (%)	44±3 (10)	40±3 a ^,^ b (9)	44±4 (10)	41±3 a ^,^ b (10)
LDH (IU/L)	108 (54–178)	2,883 (1,363–4,863) c	267 (198–556)	2,946 (1,872–7,119) b ^,^ d
	(10)	(10)	(10)	(10)
Fi (mg/dl)	164 (150–206)	60 (60–60) d	144 (60–182)	60 (60–60) b ^,^ d
	(9)	(9)	(10)	(10)
Na (mEq/L)	144 (141–149)	146 (144–152)	143 (142–147)	146 (144–147)
	(10)	(9)	(10)	(10)
K (mEq/L)	4.5±0.4	5.0±1.2	4.5±0.6	4.2±0.3
	(10)	(9)	(10)	(10)

Data are mean ± SD or median (quartiles).

a p<0.05 vs. control;

b p<0.05 vs. SP;

c p<0.01 vs. control;

d p<0.001 vs. control.

### Histological Analysis

**Figure 1:**
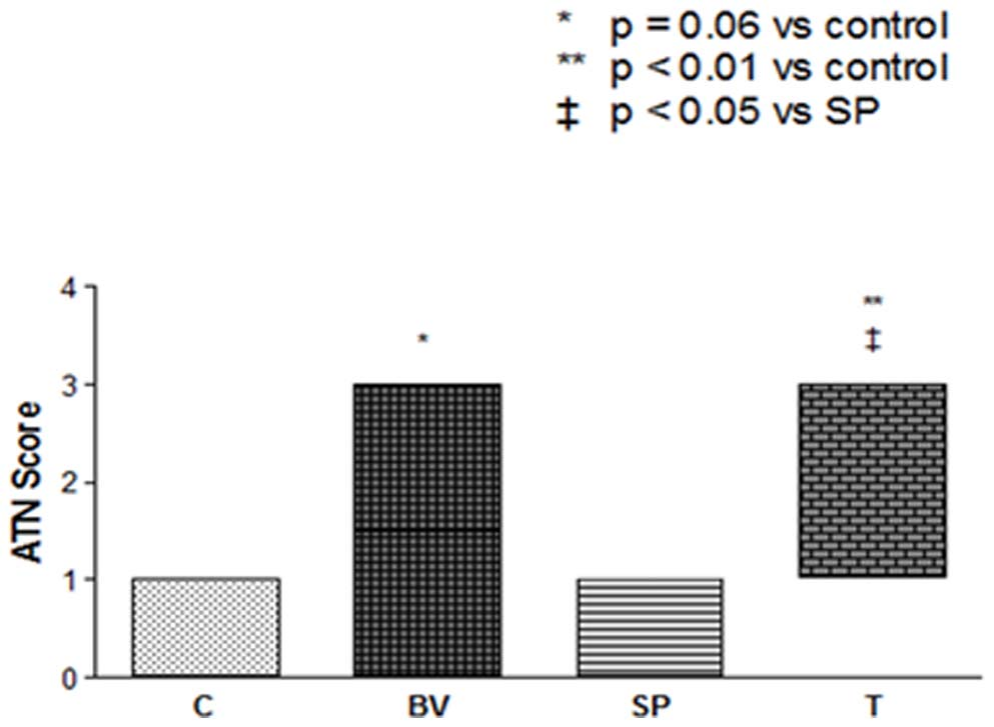
Acute tubular necrosis score in the four groups of studied rats: Control (C), *Bothrops* Venom (BV), *Schizolobium Parahyba* (SP) and treatment, which received *Schizolobium Parahyba* extract infusion immediately after *Bothrops* venom infusion (T).

Animals that received BV or BV followed by SP had higher acute tubular necrosis (ATN) scores than the control and SP alone groups. However, only the difference between the treatment group (BV followed by SP) and the control and SP groups reached statistical significance, whereas the BV group alone reached a marginal statistical significance (p = 0.06). Significant BV-induced glomerular and/or vascular injuries were not found in this study. These results are shown in Figure 1.

## Discussion

A sublethal dose of BV caused striking renal function and hemodynamic impairments, ATN, fibrinogen consumption and intravascular hemolysis without systemic blood pressure changes. These results are consistent with previous publications using the same or similar experimental BV-induced AKI models in rodents [18], [19]. In addition, BV induced a sharp and significant increase in NGAL, the urinary biomarker for renal tubular cell injury, an effect that is consistent with the functional and structural renal changes caused by BV in this study and that was not previously demonstrated in snakebite-induced AKI.

Increases in the urinary NGAL and KIM-1 levels have been consistently demonstrated in sepsis and renal ischemia and/or with nephrotoxic drug-induced AKI experimental rodent models as well as in parallel clinical situations [40], [41]. Whereas the NGAL increase occurs very early, in minutes to a few hours, following a renal insult, the KIM-1 increase occurs later, which may explain why we detected increased urinary NGAL but not KIM-1 levels in the present study after BV exposition [42].

None of the changes caused by BV were prevented by the sequential infusion of SP in an approximately 10∶1 ratio immediately after and by the same route of BV infusion. Additional experiments using 20 mg/kg of SP (provides a ratio of approximately 100∶1 for SP in relation to BV) also did not protect against the BV-induced decrease in GFR (data not shown). Of note, SP did not enhance the kidney damage caused by BV; however, SP alone caused a moderate and isolated GFR decrease in the SP group.

Mendes et al assessed the effects of SP leaf aqueous extract against the enzymatic and biological activities of *Bothrops pauloensis* and *Crotalus durissus terrificus* whole venom, along with some of their isolated toxins, namely metalloproteinase and Lys49 phospholipase A_2_ (PLA_2_) from *B. pauloensis* venom and PLA_2_ from crotoxin complex [37]. When the venoms and toxins were pre-incubated (30 min before the assays) with SP, their corresponding phospholipase A_2_, coagulant, fibrinogenolytic, hemorrhagic and myotoxic activities were significantly or completely inhibited, depending on the venom/SP ratio. However, when the hemorrhagic and myotoxic actions of the venom were assessed in vivo in mice and SP was administered 15 min later by the same route (intradermally for the hemorrhagic action tests and intramuscularly for the myotoxic action tests), there was only partial protection. The mechanisms putatively involved in the observed protection are the association of SP with venom proteins and neutralizing PLA_2_ and venom proteolytic activities [37], [38]. In a subsequent study, the same group of investigators demonstrated that flavonoids isolated from the SP leaf aqueous extract (isoquercitrin, myricetin-3-O-glucoside, catechin and gallocatechin) significantly inhibited the hemorrhagic and fibrinogenolytic activities of the metalloproteinases and the myotoxic activity of both *Bothrops alternatus* venom and Lys49 PLA_2_ from *Bothrops neuwiedi* venom [39]. However, in the present study, immediate infusion of SP after intravenous venom administration did not prevent the deleterious systemic and renal actions of BV. Our results indicate that SP is likely ineffective against BV when the venom reaches the blood stream. Considering that only when BV was pre-incubated with SP before administration was there significant protection against the snake venom or the venom fractions, the clinical usefulness of SP against the systemic effects of BV is questionable. SP or SP flavonoids might be useful as an anti-venom adjuvant therapy against BV-induced local injury.

Mendes et al assessed the acute toxic effects of intraperitoneal or oral SP aqueous extract administration in mice. Escalating the dose of SP up to 2.0 mg/g did not induce serum creatinine (SCr) changes [43]. However, SCr is neither a sensitive nor a precise marker for evaluating the renal function in mice. Using inulin clearance, we found that SP alone caused a moderate but significant decrease in GFR in rats. The renal function decline might be caused by SP-induced inhibition of the intra-renal PLA_2_ action. In fact, PLA_2_ is important for renal homeostasis in stress conditions, such as the surgical procedure the rats underwent, because PLA_2_ promotes the release of arachidonic acid, a prostaglandin precursor, from membranes [44], [45].

The present study has some limitations. Results from animal studies were not always confirmed in the clinical situation, we did not perform a formal dose response study (however even a 10 times increase in the SP dose was unable to protect against BJ-induced renal injury) and specific subfractions of SP extract, such flavonoids, were not assessed.

In conclusion, intravenous administration of SP aqueous extract immediately after intravenous BV inoculation did not protect against the systemic and renal actions of BV in rats. These results raise concerns on the clinical utility of the use of SP aqueous extract as a therapy against BV systemic effects. The local use of SP extract or SP components on a bite site might be useful as an adjuvant therapy against BV-induced local inflammatory and necrotic injury.
